# Automated Assessment of Balance Rehabilitation Exercises With a Data-Driven Scoring Model: Algorithm Development and Validation Study

**DOI:** 10.2196/37229

**Published:** 2022-08-31

**Authors:** Vassilios Tsakanikas, Dimitris Gatsios, Athanasios Pardalis, Kostas M Tsiouris, Eleni Georga, Doris-Eva Bamiou, Marousa Pavlou, Christos Nikitas, Dimitrios Kikidis, Isabelle Walz, Christoph Maurer, Dimitrios Fotiadis

**Affiliations:** 1 Unit of Medical Technology and Intelligent Information Systems Department of Materials Science and Engineering University of Ioannina Ioannina Greece; 2 Ear Institute University College London London United Kingdom; 3 Biomedical Research Centre Hearing and Deafness University College London Hospitals London United Kingdom; 4 Centre for Human and Applied Physiological Sciences King's College London London United Kingdom; 5 First Department of Otolaryngology-Head and Neck Surgery Hippokrateio General Hospital National Kapodistrian University of Athens Athens Greece; 6 Department of Neurology and Neuroscience Medical Center - University of Freiburg Faculty of Medicine, University of Freiburg Freiburg Germany; 7 Biomedical Research Institute Ioannina Greece

**Keywords:** balance rehabilitation exercises, scoring model, exercise evaluation, persuasive coaching system

## Abstract

**Background:**

Balance rehabilitation programs represent the most common treatments for balance disorders. Nonetheless, lack of resources and lack of highly expert physiotherapists are barriers for patients to undergo individualized rehabilitation sessions. Therefore, balance rehabilitation programs are often transferred to the home environment, with a considerable risk of the patient misperforming the exercises or failing to follow the program at all. Holobalance is a persuasive coaching system with the capacity to offer full-scale rehabilitation services at home. Holobalance involves several modules, from rehabilitation program management to augmented reality coach presentation.

**Objective:**

The aim of this study was to design, implement, test, and evaluate a scoring model for the accurate assessment of balance rehabilitation exercises, based on data-driven techniques.

**Methods:**

The data-driven scoring module is based on an extensive data set (approximately 1300 rehabilitation exercise sessions) collected during the Holobalance pilot study. It can be used as a training and testing data set for training machine learning (ML) models, which can infer the scoring components of all physical rehabilitation exercises. In that direction, for creating the data set, 2 independent experts monitored (in the clinic) 19 patients performing 1313 balance rehabilitation exercises and scored their performance based on a predefined scoring rubric. On the collected data, preprocessing, data cleansing, and normalization techniques were applied before deploying feature selection techniques. Finally, a wide set of ML algorithms, like random forests and neural networks, were used to identify the most suitable model for each scoring component.

**Results:**

The results of the trained model improved the performance of the scoring module in terms of more accurate assessment of a performed exercise, when compared with a rule-based scoring model deployed at an early phase of the system (k-statistic value of 15.9% for sitting exercises, 20.8% for standing exercises, and 26.8% for walking exercises). Finally, the resulting performance of the model resembled the threshold of the interobserver variability, enabling trustworthy usage of the scoring module in the closed-loop chain of the Holobalance coaching system.

**Conclusions:**

The proposed set of ML models can effectively score the balance rehabilitation exercises of the Holobalance system. The models had similar accuracy in terms of Cohen kappa analysis, with interobserver variability, enabling the scoring module to infer the score of an exercise based on the collected signals from sensing devices. More specifically, for sitting exercises, the scoring model had high classification accuracy, ranging from 0.86 to 0.90. Similarly, for standing exercises, the classification accuracy ranged from 0.85 to 0.92, while for walking exercises, it ranged from 0.81 to 0.90.

**Trial Registration:**

ClinicalTrials.gov NCT04053829; https://clinicaltrials.gov/ct2/show/NCT04053829

## Introduction

Balance rehabilitation is essential evidence-based treatment for patients with balance disorders, especially when they are at risk of falls [1]. However, it is not feasible or economically affordable to provide patients with in-hospital sessions involving a dedicated clinician for all rehabilitation sessions required [2]. Physiotherapy health services are provided in hospitals or outpatient clinics, with assessment sessions conducted in-person by clinicians, followed by unsupervised rehabilitation sessions in the patients’ homes (eg, Otago Exercise Program [3]). Research groups and published reports have shown that more than 90% of all treatments are home based [4]. According to these procedures, patients are asked to report their daily activities related to the instructed exercises and actions at home. Actual progress evaluation is performed during visits to the physician [5]. Low patient motivation and adherence to the appropriate rehabilitation exercise programs have been reported, and these consequently prolong treatment times and impose higher health care costs [6]. While various factors have been identified that contribute to low compliance, lack of continuous feedback is an important factor, and accurate monitoring of patient exercises by medical professionals in a home environment is considered essential [7,8].

A typical home-based rehabilitation exercise program (with no digital tools integrated) is based on a handbook of instructions and directions about the frequency, intensity, and correct performance of physiotherapy exercises [8]. Yet, such programs do not always ensure the full recovery of patients, as compliance rates are low [9]. In turn, activity recognition and evaluation have received increasing attention in the fields of machine learning (ML) and computer vision. Especially during the COVID-19 outbreak, the need for enhancing typical home-based rehabilitation programs with sensing devices and virtual reality interaction has substantially increased [10].

Activity recognition approaches use sensing devices to collect appropriate signals and infer the performed activity. Sensing devices vary in complexity and cost, and include video sensors, inertial measurement units, and pressure sensors. Motion analysis based on video signals explores various representations, like skeleton extraction and space-time volume. While many visual techniques have been used in recent decades, large differences in anatomy, human occlusion, and changes in perspectives often limit the capacity of the proposed models to correctly assess the performance of an exercise. Sensing technology (apart from video) has made significant progress during the last decade, especially with low-power devices, wireless communication, high computational capacity, and data processing [11]. Wearable sensors can be integrated in clothes, strips, mobile devices, and smartwatches [12]. It is important to mention that the assessment of balance rehabilitation exercises requires accurate identification of specific movements and kinematics during the execution of the exercise (eg, head movement speed and direction, and chest flexion).

In contrast to the pure recognition of an activity, in rehabilitation programs especially, the evaluation of exercise execution is of paramount importance. This is especially significant for recovery, as it demonstrates whether the patient can perform the prescribed process [13]. During the last few years, several approaches for exercise evaluation have been proposed. In a previous study [14], a smart sensor–based rehabilitation exercise recognition and evaluation system using a deep learning framework was proposed. The main limitation was data synchronization from several sensors related to activity recognition. In similar approaches, the collected data include noise and vary when different people perform the same activity [15]. Furthermore, a state probability transition is proposed to show the transition likelihoods among states to capture the hidden states of sensory data. To test rehabilitation activities, a special matrix has been introduced, and the learned classifier has been used to identify the best features of every class at various levels. The scoring functions are given for the (0-1) range of the output values tested. To train the proposed deep neural networks in rehabilitation, the resulting movement quality scores have been used [16].

A previous study [17] proposed the hidden semi-Markov model for the assessment of rehabilitation exercises. The method extracts clinically related motion features from an RGB-D camera’s skeleton and proposes an abstract representation of the subject. The effectiveness of the proposed solution has been assessed by analyzing the correlation between both a clinical evaluation and dynamic time-warping algorithms. Additionally, a previous study [18] proposed the multi-path convolutional neural network (CNN) for the recognition of rehabilitation exercises. The results of the classification accuracy in the relative experiments showed that a multi-path CNN is highly efficient for sensor data acquisition. In another study [19], a deep learning–based framework for rehabilitation exercise assessment was introduced. The main modules of the system were the calculation of metrics for the quantity of motion output, the scoring of performance assessment functions for numerical motion quality ratings, and deep neural network models for quality regression of input motion through supervised learning. A previous survey [20] suggested sensor-based activity recognition by deep learning. More specifically, the survey [20] presented the recent progress in sensor-based recognition in a deep learning model, where the authors summarized the current literature (deep models and sensory techniques). Finally, a previous paper [21] assessed physical activity recognition and monitoring using Internet of Things and presented a systematic review of existing studies.

The recent development of deep learning allows high-level automated feature extraction to achieve promising performance in numerous areas [22]. Deep learning approaches for sensor-based activity recognition have been widely adopted. Further, deep learning can greatly reduce the strain on features and can acquire much higher and meaningful features by training a neural end-to-end network. Furthermore, the deep network structure facilitates uncontrolled and incremental learning. However, compared with supervised learning approaches, deep learning models require a substantially large amount of data, which are, in general, not available in the physiotherapy domain. Thus, bearing in mind the individualities of the physiotherapy exercises, feature engineering is mandatory for each specific exercise.

**Figure 1:**
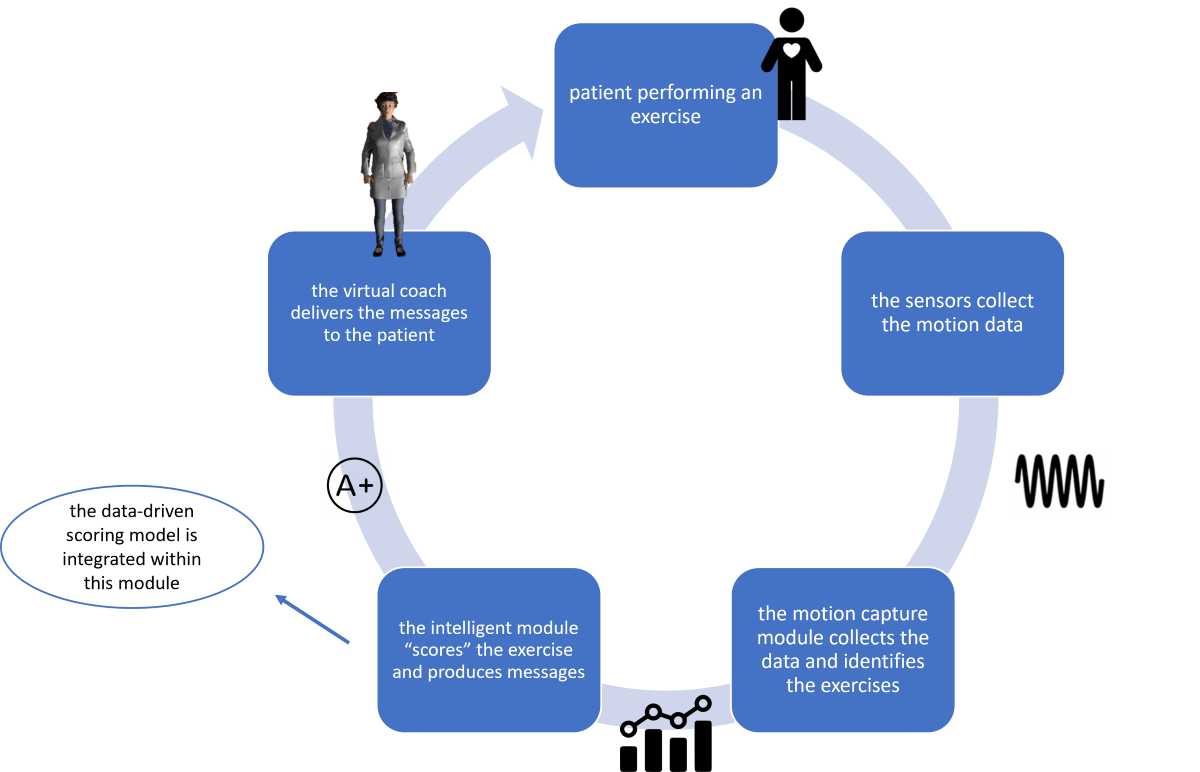
Virtual coaching closed-loop interaction. The proposed model is integrated into the “intelligent” module of the virtual coaching system.

In our previous work [23], we have proposed a framework for managing a balance physiotherapy program at home. This framework (Figure 1), which has been designed and developed within the Holobalance project, comprises a holographic virtual coach, presented to the patient through an augmented reality system, a motion sensing platform, and a smart engine, which assesses in real time the exercise performance. Details on the overall architecture of the system can be found elsewhere [24,25]. The technology supporting the virtual coach augmented reality module is described in several studies (eg, [26]), where information regarding augmented reality systems in rehabilitation systems can be found.

The aim of this study was to design, implement, test, and evaluate a scoring model for the accurate assessment of balance rehabilitation exercises, based on data-driven techniques. More specifically, this work presents an improved model for the offline scoring function, which is not based on the knowledge-based model that was used previously [23], but is based on a data-driven model with the capacity to predict with higher accuracy the score of a performed exercise. As it is of paramount importance for a closed-loop persuasive system to correctly evaluate the performance of an exercise, the proposed scoring model is expected to provide more robust and reliable feedback to the overall system’s reasoning engine.

## Methods

### Ethics Approval

This study has received institutional ethics approvals in Germany/Freiburg (reference: 265/2019) and Greece/Athens (reference: 9769/24-6-2019).

### Study Design

A pilot study with 20 participants was conducted with the aim to collect the appropriate data set to develop the scoring model. After 1 dropout, 19 patients followed an 8-week balance rehabilitation program, according to the protocol described previously [27] at 2 pilot sites. Participants were elderly individuals who had experienced at least one fall during the last year. They were all informed about the context of the study and volunteered to participate, after providing their written consent regarding the willingness to use the Holobalance system in the clinic and to have their data recorded and used for research purposes.

While the Holobalance system is designed for home use, it was installed in a clinic setup to test safety and to collect the necessary data. After recruitment of the patients, functional and cognitive assessments were performed based on the Mini-Balance Evaluation Systems Test (MINIBEST), Functional Gait Assessment (FGA), Falls Efficacy Scale International (FES-I), Montreal Cognitive Assessment (MoCA), World Health Organization Disability Assessment Schedule (WHODAS), and Activities-Specific Balance Confidence Scale (ABC), as per the clinical study protocol [27]. It is important to mention that while both the FES-I and ABC attempt to infer similar information about the patient, their outputs are not fully correlated [28]. Demographic data as well as the distribution of the tests are presented in Table 1. According to FGA results, the population of this study had mild cognitive impairment [1].

**Table 1:** Study participant details.

Variable	Pilot site			Total value
	Athens	Freiburg		
Participants, n	14	5	19	
Age (years), median (IQR)	64.5 (15.5)	72.0 (4.0)	68.0 (11.0)	
Height (cm), median (IQR)	157.5 (11.8)	170.0 (2.0)	160.0 (16.5)	
Weight (kg), median (IQR)	67.0 (21.5)	69.0 (8.0)	69.0 (21.0)	
Male gender, %	7.14	40.00	15.79	
Mini-Balance Evaluation Systems Test score (range^a^ 0-28), median (IQR)	21.5 (6.0)	21.0 (1.0)	21.0 (5.5)	
Functional Gait Assessment score (range^a^ 0-30), median (IQR)	21.0 (5.0)	22.0 (3.0)	21.0 (5.5)	
Falls Efficacy Scale International score (range^a^ 16-64), median (IQR)	27.5 (9.25)	19.0 (8.0)	27.0 (8.5)	
Montreal Cognitive Assessment score (range^a^ 0-30), median (IQR)	25.5 (3.75)	27.0 (4.0)	26.0 (4.0)	
World Health Organization Disability Assessment Schedule score (range^a^ 100-0), median (IQR)	23.0 (24.5)	17.0 (21.0)	17.0 (22.0)	
Activities-Specific Balance Confidence Scale score (range^a^ 0-100), median (IQR)	76.9 (20.3)	87.5 (15.0)	82.5 (19.9)	

^a^For the score range a-b, “a” represents no disability and “b” represents the highest disability.

### Data Set

The participants, following the balance rehabilitation program prescribed by their physicians, performed a set of exercises during 16 sessions (2 sessions per week). During each session, a set of exercises was performed according to the program. The number of exercises per session varied from 3 to 8. Participants were instructed to execute the exercises at a self-paced rate (frequency and velocity of the movements) that would make them feel comfortable, avoiding any symptoms. As the sessions progressed, the aim of the program was to increase these metrics.

The performed exercises (with the relative progression levels for each exercise), which are described in a previous paper [27], were grouped into 9 classes, according to the kinematic characteristics of each exercise. The rehabilitation protocol included 3 types of exercises (sitting exercises, standing exercises, and walking exercises). More specifically, there were 3 sitting exercises with 3 progression levels (in terms of intensity and complexity), 4 standing exercises with 4 progression levels, and 3 walking exercises with 3 progression levels (Table 2). The exercises were designed under the rationale of progressiveness of difficulty, including both simple and complex tasks, aiming for head-eye-hand coordination through multisensory rehabilitation exercises. As reported previously [29], the system is acceptable by end users and is feasible for use in hospital and home environments.

The data set was collected from April 2020 to June 2021. In total, 1313 exercises were recorded. Table 3 summarizes the collected annotated exercises.

**Table 2:** Description of the available rehabilitation exercises offered within the Holobalance intervention protocol (adapted from Liston et al [27], which is published under Creative Commons Attribution 4.0 International License [30]).

Exercise type	Exercise description
Sitting 1: Yaw	Perform head rotations of 30 degrees in the yaw plane (ie, left-right) while sitting, aiming at enhancing gaze stability.
Sitting 2: Pitch	Perform head rotations of 30 degrees in the pitch plane (ie, up-down) while sitting, aiming at enhancing gaze stability and improving common vestibular symptoms such as dizziness, swimminess, and light-headedness.
Sitting 3: Bend over	Bend as if to pick up an object off the floor from the sitting position and return to the upright position, aiming at improving functional activities of daily living (ADL) tasks and mitigating vestibular symptoms if provoked through practice.
Standing 1: Maintain balance	Maintain balance while standing up and remain in the proper position, aiming at improving postural alignment and standing ability with a smaller base of support.
Standing 2: Maintain balance on foam	Maintain balance as in standing exercise 1 while standing on a cushion and remain in the proper position, aiming at promoting sensory reweighting.
Standing 3: Bend over and reach up	Bend over bringing the chin to the chest, return the head to the normal upright position on coming up, and reach up while slightly tilting the head back, aiming at improving functional ADL tasks and dizziness.
Standing 4: Turn	On site, turn to face the opposite direction (ie, 180° turn), aiming at improving functional ADL tasks and dizziness.
Walking 1: Walk to horizon	Walk across the room (back and forth) in a straight path while looking at the horizon, aiming at promoting a normal gait pattern. Minimum space of 2 meters.
Walking 2: Walk & yaw	Walk across the room (back and forth) in a straight path while turning the head left and right, aiming at improving gaze stability while walking and functional ADL walking tasks. Minimum space of 2 meters. Yaw movement as in sitting exercise 1.
Walking 3: Walk & pitch/V-shape	Walk across the room (back and forth) in a straight path while turning the head up and down, and with V-shaped movement, aiming at improving gaze stability while walking and functional ADL walking tasks. Minimum space of 2 meters. Yaw and pitch movements as in sitting exercises 1 and 2.

**Table 3:** Exercises according to the type and progression level (N=1313).

Exercise type			Value, n		Exercise progression
**Sitting exercise**			514		
	Sitting exercises 1 and 2	347		All progression levels	
	Sitting exercise 3	167		All progression levels	
**Standing exercise**			530		
	Standing exercises 1 and 2	312		All progression levels	
	Standing exercise 3	97		Progression levels 0 and 1 included 46; progression level 2 included 19; progression level 3 included 32	
	Standing exercise 4	121		All progression levels	
**Walking exercise**			269		
	Walking exercise 1	87		All progression levels	
	Walking exercises 2 and 3	182		All progression levels	

During the execution of the exercises, a physiotherapist monitored the patient and scored patient performance using a scoring rubric that included 4 components (frequency, amplitude, velocity, and symmetry) for the sitting and standing exercises and an additional component (gait quality) for the walking exercises. For exercises with complex kinematic characteristics, additional components were considered in the scoring. For example, if an exercise included movement of the head and walking, rubric components for head movement and for gait quality were included in the scoring process.

More specifically, for sitting exercises, frequency referred to the number of head rotations (eg, in the yaw plane for sitting exercise 1) per second, while amplitude referred to the degree of head turn from the upfront position to the extreme points of the movement. Additionally, velocity referred to the number of seconds a patient needed to perform a movement. This metric differs from frequency, as patients usually paused for some seconds between exercise movements, especially for complex ones like sitting exercise 3.

For each component, a score from 0 to 3 was given, with a score of 0 representing the noncompletion of the exercise. On top of the rubric components, a total score for each exercise was calculated a posteriori as the average of all components (N) of an exercise.



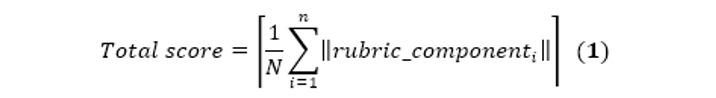



The proposed scoring model infers the score for all the involved components of an exercise, as well as the total score, which is mainly required to provide input to adjacent modules of the persuasive coaching system.

All patients undertook training sessions to get familiarized with the system. In addition, the session physiotherapists provided specific instructions for the correct execution of the exercises to the patients, in terms of timing and kinesiology. As described previously [23], these instructions were used to create the knowledge-based scoring model of the system.

A subset of the data set described in Table 3 was annotated by 2 physiotherapists, who monitored the patients during the execution of the exercises. More specifically, 38 sessions from 4 patients, which included 90 sitting exercises, 78 standing exercises, and 59 walking exercises, were scored by 2 independent evaluators to assess the interobserver variability of the annotation process. This resulted in 665 annotated scores for the different components of the scoring rubric.

### Metrics and Analytics

**Figure 2:**
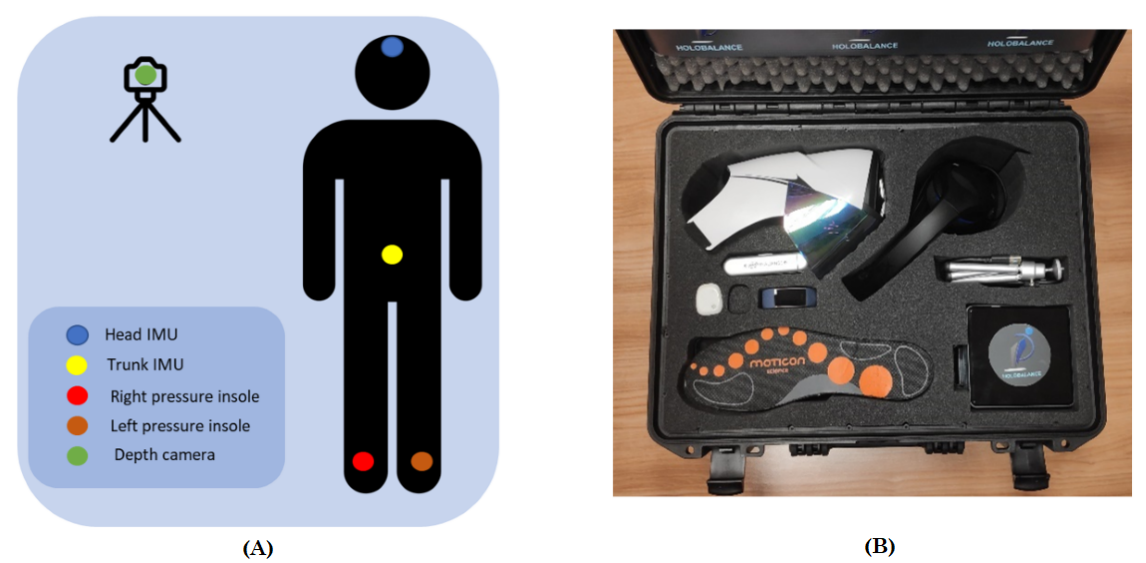
The Holobalance system. (A) Sensor positioning in the Holobalance system. (B) Devices of the Holobalance system. IMU: inertial measurement unit.

As presented previously [23], based on a set of sensing devices (Figure 2), the system collected temporal signals and processed them by extracting specific kinematic metrics, which were translated to exercise analytics. These analytics, along with the knowledge-based scoring model presented previously [23], were used as features in the ML models used to constitute the scoring model. Table 4 summarizes the extracted features, which were used as inputs for the ML models. The build prototype of the home-based system, including all the sensing devices, the head-mounted display, and the processing unit, costs approximately €4800 (US $4850) (Figure 2).

The knowledge-based exercise score model (kb_score), mentioned in Table 4, refers to a rule-based model that attempts to assess the performance of an exercise based on the values of the captured motion analytics. More specifically, a group of experts established the acceptable range for each of the motion analytics (eg, 30 degrees for the head movement in sitting exercise 1). Based on these ranges, the knowledge-based model calculates the proportion of time a patient performs within these ranges, as well as how close the patient comes to the optimal range, and outputs the final kb_score. For assessing balance, sway, and stability, posture and trunk_sway metrics (Table 4) have been used.

**Table 4:** Input features for training the machine learning models.

Feature	Description
kb_score	Knowledge-based exercise score as proposed previously [27]
head_movement_speed	Number of head rotations per second (mean and standard deviation) in the yaw and pitch planes
head_movement_range	Range of head rotations (mean and standard deviation) in the yaw and pitch planes
posture	Angle of the torso (sitting and standing)
trunk_sway	Mean and standard deviation of trunk sway
gait_parameters	Center of pressure on both feet (mean distance covered by the center of pressure and standard deviation per gait cycle); double support time (mean value and standard deviation per gait cycle); single support time (mean value and standard deviation per gait cycle); step duration (mean value and standard deviation per gait cycle); stride duration (mean value and standard deviation per gait cycle); cadence (mean value and standard deviation per gait cycle)

### Scoring Model

**Figure 3:**
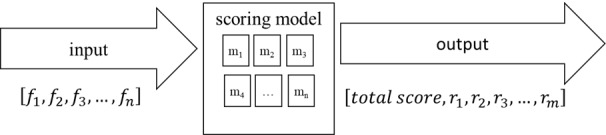
The scoring model.

The proposed data-driven exercise scoring model uses as inputs the analytics described in Table 4 and outputs a scoring vector for each exercise, as presented in Figure 3. More specifically, *f_i_* refers to the features that describe the motion and movement of a patient during the performance of an exercise, while *r_i_* refers to each one of the evaluation components (frequency, amplitude, velocity, and symmetry), as expressed in each different exercise. Finally, *total score* refers to an overall assessment of the exercise. As the importance of the input features varies for the different exercise categories (Table 3), a separate model for each one of these groups of exercises and progressions has been developed and incorporated in the final scoring model.

Aiming to identify the most relevant ML model for each rubric component (and for the total score), a set of ML models was assessed for each one of the components. The considered models were k-nearest neighbors (kNN) [31], support vector machines (SVMs) [32] (with both lineal and radial basis function), Gaussian process [33], random forests [34], neural networks [22], naïve Bayes [35], and AdaBoost [36]. These specific models were selected as they have been used in a wide set of similar data-driven problems [37].

For standing exercise 3, it was required to consider different models for different progressions owing to different kinematic characteristics in its progressions. This resulted in relatively small data sets for these cases. For this, the SMOTE (synthetic minority oversampling technique) algorithm [38] was used to oversample the collected instances in order to obtain the necessary data to train the ML models.

**Figure 4:**
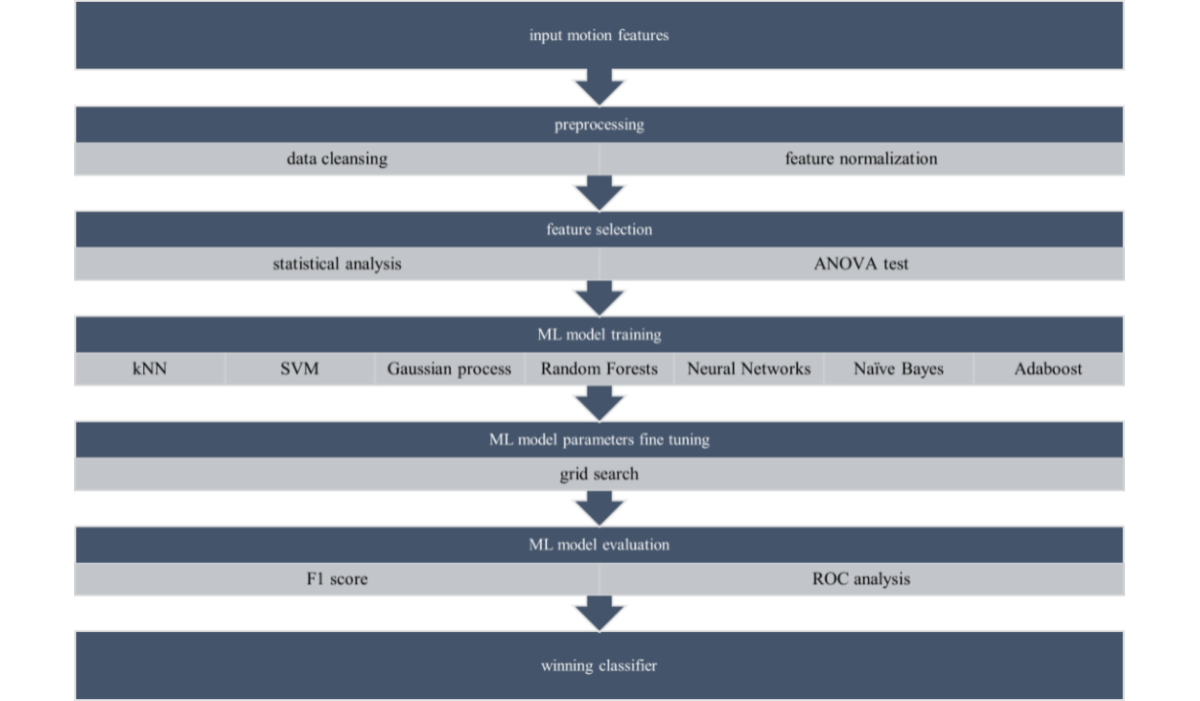
Machine learning (ML) model training approach. kNN: k-nearest neighbors; ROC: receiver operating characteristic; SVM: support vector machine.

The approach followed during the training of the ML models is summarized in Figure 4. More specifically, the first step was to identify data inconsistencies, like missing values, and remove them from the data set. Afterwards, min-max feature normalization was applied, aiming to improve the training process of the ML models. The next step involved an iterative process of training different ML models and evaluating them. For each model, an intermediate step for fine-tuning each parameter was applied, mainly using the grid search approach. Finally, the winning classifier for each model was selected, based on F1-score and receiver operating characteristic analysis results.

### Deployment Details: Integration

The winning classifiers were implemented under Python 3.8, using the scikit-learn 0.24 library. As soon as the system identifies the performed exercise, the appropriate classifier is invoked and the score of the exercise is inferred. This is now part of the Holobalance system, which is currently under evaluation.

## Results

### Overview

Within this section, the results of the training and evaluation of the ML models for each component of the scoring rubric are presented. All models were evaluated by applying a 10-fold cross-validation process and assessing the macro-average accuracy of the models. The training and testing data sets for each fold were created under an 80/20 ratio.

### Interobserver Variability

As already mentioned earlier, almost 17.3% of the recorded exercises were scored by 2 observers to assess the interobserver variability of the annotation process. The results of this procedure are presented in Table 5. The selected evaluation metric is Cohen kappa coefficient [39], which is calculated as follows:



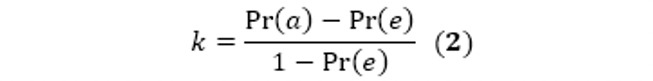



where Pr(*a*) is the relative observed agreement among raters and Pr(*e*) is the hypothetical probability of chance agreement, using the observed data to calculate the probability of each observer randomly seeing each category. If the raters are in complete agreement, then *k*=1. If there is no agreement between the raters other than what would be expected by chance (as given by Pr(*e*)), then *k*=0.

**Figure 5:**
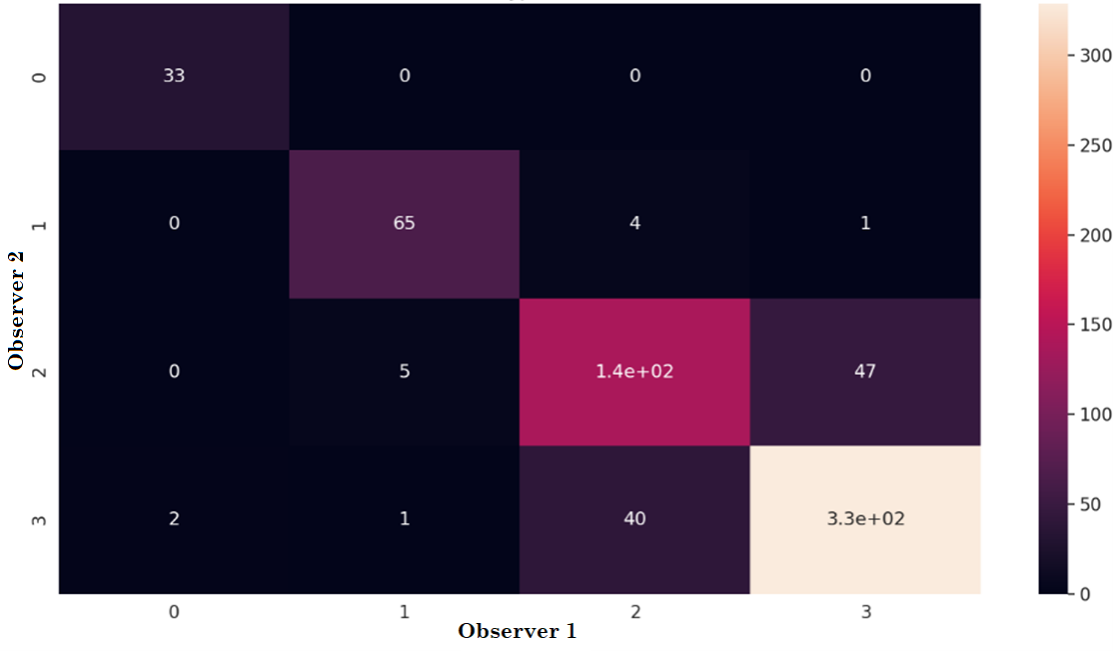
Confusion matrix. All types of exercises (N=665) in the annotation process of 2 observers.

From a previous study [40], it can be concluded that the agreement of the observers was “good,” allowing the use of the collected data set to train reliable ML models. Figure 5 presents the confusion matrix of the annotation process (please see Multimedia Appendix 1 for more details).

**Table 5:** Results of interobserver variability per exercise type.

Exercise type	k statistic
All exercises	0.75
Sitting exercises	0.68
Standing exercises	0.79
Walking exercises	0.75

### Classification Results of Each Model

As mentioned earlier, an ML model for each component of the scoring rubric was trained and evaluated. The results are presented in Table 6, where the macro-average accuracy has been provided, along with the winning classifier for each model. The results below present a set of 40 trained classifiers, which finally constitute the system’s scoring model. More detailed results for the classification models are presented in Multimedia Appendix 1.

For the sitting and standing exercises, it can be observed that the Gaussian process is the most relevant classifier, most probably because the number of features was lower compared with that for the walking exercises. Additionally, the low number of input features was correlated with higher accuracy results, which was expected. Thus, the accuracy for sitting exercises 1 and 2 was almost 90%, while that for walking exercises 2 and 3 dropped to slightly higher than 80% (Table 6). Finally, for the total score, the random forest classifier outperformed the rest of the models for 2 exercise subgroups, while kNN and linear SVM outperformed for 1 subgroup.

**Table 6:** Macro accuracy results of the winning classifiers for each of the considered models.

Exercise type	Macro accuracy/winning classifier						
	Total score	Component 1	Component 2	Component 3	Component 4	Component 5	Component 6
Sitting 1 and sitting 2	0.90/Gaussian process	0.88/Gaussian process	0.90/kNN^a^	0.89/Gaussian process	N/A^b^	N/A	N/A
Sitting 3	0.87/Gaussian process	0.86/Neural network	0.91/Gaussian process	N/A	N/A	N/A	N/A
Standing 1 and standing 2	0.85/Gaussian process	0.83/Gaussian process	0.86/Gaussian process	N/A	N/A	N/A	N/A
Standing 3 (progressions 0-1)	0.91/kNN	0.91/Gaussian process	0.92/Gaussian process	0.89/kNN	0.90/Random forest	N/A	N/A
Standing 3 (progression 2)	0.87/SVM^c^ (linear)	0.89/Gaussian process	0.90/Naïve Bayes	0.88/Random forest	0.91/kNN	N/A	N/A
Standing 3 (progression 3)	0.91/Random forest	0.90/AdaBoost	0.88/Neural network	0.86/kNN	0.89/kNN	N/A	N/A
Standing 4	0.92/Gaussian process	0.86/Gaussian process	0.88/Gaussian process	0.80/kNN	N/A	N/A	N/A
Walking 1	0.90/Random forest	0.81/Gaussian process	0.85/Random forest	0.92/Random forest	N/A	N/A	N/A
Walking 2 and walking 3	0.81/kNN	0.74/kNN	0.75/SVM (linear)	0.78/SVM (RBF^d^)	0.71/kNN	0.75/SVM (RBF)	0.75/kNN

^a^kNN: k-nearest neighbors.

^b^N/A: not applicable.

^c^SVM: support vector machine.

^d^RBF: radial basis function.

### Overall Results: k-Statistic Analysis

Table 7 presents the overall results of the classification models for each individual exercise and the progression levels. In the same table, comparisons of interobserver variability, and the variability among observer 1 and the trained ML models are provided, which were performed on the testing data sets of each model. In addition, the previously used knowledge-based model [23] was compared with the annotations of the first observer.

Based on the results, the proposed framework’s performance was similar to interobserver variability, thus constituting a reliable model for automated scoring of balance physiotherapy exercises. More specifically, the variability for the sitting exercises was almost identical, while there was a drop of 0.02 for the standing exercises. Finally, for the walking exercises, the decrease in the k-statistic was 0.04, which was justified due to the increased complexity of the relative exercises and the increased input features for the classification problems in these specific exercises.

When compared with the knowledge-based scoring model, the improvement in the agreement was substantial (15.9% for sitting exercises, 20.8% for standing exercises, and 26.8% for walking exercises for the k-statistic). This improvement enables the system to effectively deduce the performance of the patient, and thus, the system can not only correctly inform the clinician about the patient’s status, but also enable them to design/choose correctly future rehabilitation programs.

**Table 7:** Overall classification accuracy and k-statistic analysis.

Exercise type		Total score (model)	k statistic (interobserver variability)	k statistic (observer 1 – ML^a^ model)	k statistic (observer 1 – knowledge-based model)
**Sitting**			0.68	0.69	0.58
	Sitting exercises 1 and 2	0.90 (Gaussian process)			
	Sitting exercise 3	0.86 (Gaussian process)			
**Standing**			0.79	0.77	0.61
	Standing exercises 1 and 2	0.853 (Gaussian process)			
	Standing exercise 3 (progression level 0-1)	0.912 (kNN^b^)			
	Standing exercise 3 (progression level 2)	0.8736 (SVM^c^ linear)			
	Standing exercise 3 (progression level 3)	0.905 (random forest)			
	Standing exercise 4	0.918 (Gaussian process)			
**Walking**			0.75	0.71	0.52
	Walking exercise 1	0.899 (random forest)			
	Walking exercises 2 and 3	0.813 (kNN)			

^a^ML: machine learning.

^b^kNN: k-nearest neighbors.

^c^SVM: support vector machine.

## Discussion

### Principal Findings

The proposed set of ML models can effectively score the balance rehabilitation exercises of the Holobalance system. The models had similar accuracy in terms of Cohen kappa analysis, with interobserver variability, enabling the scoring module to infer the score of an exercise based on the collected signals from sensing devices. More specifically, for the sitting exercises, the scoring model had high classification accuracy, ranging from 0.86 to 0.90. Similarly, for the standing exercises, the classification accuracy ranged from 0.85 to 0.92, while for the walking exercises, it ranged from 0.81 to 0.90. From the obtained results, we observed that the lowest classification accuracies were related to the most complex exercises, in terms of required movements. While this result was anticipated, it is interesting that the same exercises also presented the highest interobserver variability, revealing that objectively scoring a complicated exercise is not a trivial task, even for expert physiotherapists. This is clearly reflected by the k-statistic analysis for almost all different exercise types. It is also important to mention that most of the misclassifications involved classes 2 and 3, meaning that poor performance (classes 0 and 1) and adequate performance (classes 2 and 3) can be assessed more accurately, by both the experts and the scoring model.

### Comparison With Prior Work

The first version of the scoring module was built upon medical knowledge extracted by a group of experts [27]. The main drawback of this model was that it could not capture all possible states of a patient during the execution of a balance rehabilitation exercise. Thus, it failed in various situations to correctly grade the patient. The proposed data-driven model significantly improves the accuracy for the performed exercises, increasing the k-statistic by 0.11 for sitting exercises, 0.16 for standing exercises, and 0.19 for walking exercises. It was noticeable that a more complex exercise was associated with higher improvement.

### Strengths

The novelty of this work can be summarized in 2 main remarks. First, an annotated data set of sensor signals during the performance of about 1300 exercise sessions from 19 patients, along with the scoring of the exercises from an expert, was created. To the best of our knowledge, no such data set has been reported in the literature. Second, a scoring module, which includes several ML-supervised learning models, was developed and tested. The results clearly indicate that the proposed model appears to have similar predicting capacity considering the interobserver variability of experts who annotated the ground-truth data set.

Within the context of the Holobalance system, the capacity of the scoring module obviously enables correct exercise assessment in a rehabilitation program, as a physician can monitor the performance and progress of a patient and adopt the program accordingly. This assessment has a 2-fold advantage. First, the physiotherapist managing the patient is properly informed about the performance of the patient; thus, the next rehabilitation phases are designed based on objective information, which avoids the bias of self-reported results. Second, the virtual coach interaction with the patient is based on accurate scores, which facilitates realistic interaction with the system. More specifically, the exercise progression module is based on the scores produced by the scoring module to correctly assess whether a patient should progress to the next level of an exercise. As discussed earlier, each exercise is administered at different levels in terms of difficulty, speed, and repetitions. Hence, the high accuracy of the scoring module enables the proper function of the exercise progression module. Additionally, the scoring module can be used for “red flagging” patients with very low performance and adherence early, thus allowing the physiotherapist to alter the rehabilitation approach. These aspects have a direct impact on the safe and effective execution of rehabilitation programs in home environments.

It is also important to stress that compared with other scoring models (eg, [41] and [42]), the output of the proposed model assesses not the recognition of the performed exercise but the quality of the performance of the exercise, a crucial aspect in the assessment of a rehabilitation program. By providing a high-accuracy exercise assessment model, as the one presented, virtual coaching systems can be equipped with the capacity to interact with patients using personalized context, thus enriching user experience.

Besides the value of a reliable scoring module within a persuasive coaching system like Holobalance, this module can be used independently as a separate module in clinical practice. One of the most important uses is objective baseline assessment of a patient, as it can support clinicians in objectively evaluating the baseline of a patient when performing an exercise during the first clinic visit. Additionally, the analysis for building the scoring module, especially the feature statistics analysis, can contribute to the design of new balance rehabilitation exercises targeting mainly the metrics that appear to have an important contribution to the score of an exercise, while eliminating aspects and kinematics related to metrics of low importance to the model. Furthermore, the scoring module can support patients who require long-term monitoring, especially those with degenerative neurological conditions, such as ataxia or dementia, which require long-term rehabilitation and monitoring for maintenance purposes. Moreover, a reliable scoring and assessment module can facilitate the education of novice physiotherapists and physicians, enabling them to better understand the needs of different clinical populations. Finally, within the research context, the sensor-based information from this model could be used as a biomarker to monitor populations of interest over the long term (such as older adults or patients with cognitive impairments) for the early prediction of the risk of falls and early prediction of cognitive decline.

### Limitations

Regarding the limitations of the proposed model, a major drawback is that the model requires knowledge of the type of exercise to assess the score for the exercise. In other words, the proposed scoring model does not have the capacity to recognize the exercise, limiting its usage to only rehabilitation programs with predefined exercise sets. Additionally, the size of the collected data set did not allow us to test deep learning models, which might show higher classification accuracies.

### Future Directions

Regarding the future directions related to the scoring model, we anticipate to incorporate motion recognition algorithms, enabling the module to infer which exercise is performed. This will allow the module to support free-program exercise sessions. Finally, deploying the module to more sites will allow us to extend the exercise data set, which will provide wider validation to the proposed solution and help in the use of deep learning models, if the volume of data is adequate.

## Appendix

Multimedia Appendix 1 Additional study results.

## Abbreviations

ABC: Activities-Specific Balance Confidence Scale
CNN: convolutional neural network
FES-I: Falls Efficacy Scale International
FGA: Functional Gait Assessment
kNN: k-nearest neighbors
ML: machine learning
SVM: support vector machine

## Data Availability

The data sets generated during or analyzed during this study are available from the corresponding author on reasonable request.
