# Supplementary material for: Automated Assessment of Balance Rehabilitation Exercises With a Data-Driven Scoring Model: Algorithm Development and Validation Study
Source: JMIR Rehabil Assist Technol. 2022 Aug 31;9(3):e37229. doi: 10.2196/37229 (PMC9475421; doi:10.2196/37229)
Supplement: Multimedia Appendix 1 [file rehab_v9i3e37229_app1.docx]

**Multimedia Appendix 1**

Figure S1 provides the annotations per exercise type by the two observers.


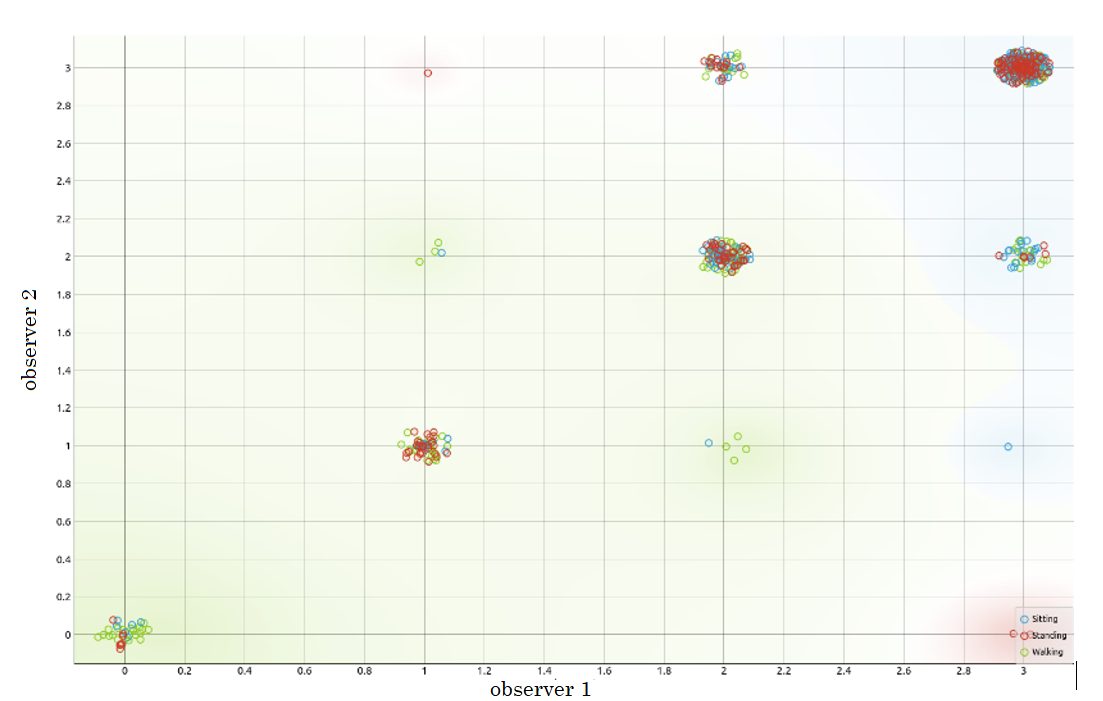


Figure S1: Comparison between observers’ scores per exercise type.

Detailed results on the evaluation of different machine learning models are presented in Table S1 to Table S9. For each scoring component and for the total score, the winning classifier is highlighted. Additionally, the ROC analysis for each tested classifier is presented Figure S2.

Table S1: Evaluation of different classifiers for sitting exercises 1 and 2.

| **Model** | **accuracy (macro-average)** | | | |
| --- | --- | --- | --- | --- |
|  | **Total Score** | **component 1** | **component 2** | **component 3** |
| **kNN** | 0.869144 | 0.848254 | **0.900616** | 0.851697 |
| **SVM (linear)** | 0.662377 | 0.702186 | 0.727338 | 0.555824 |
| **SVM (RBF)** | 0.670337 | 0.687912 | 0.693093 | 0.584518 |
| **Gaussian Process** | **0.899743** | **0.879096** | 0.893922 | **0.893810** |
| **Random Forest** | 0.667099 | 0.696703 | 0.728553 | 0.546825 |
| **Neural Network** | 0.767660 | 0.708767 | 0.805423 | 0.564579 |
| **Naïve Bayes** | 0.596459 | 0.311294 | 0.567169 | 0.498327 |
| **AdaBoost** | 0.511816 | 0.356618 | 0.487331 | 0.508400 |

Table S2: Evaluation of different classifiers for sitting exercise 3.

| **model** | **accuracy (macro-average)** | | |
| --- | --- | --- | --- |
|  | **Total Score** | **component 1** | **component 2** |
| **kNN** | 0.808036 | 0.819118 | 0.861111 |
| **SVM (linear)** | 0.468588 | 0.676923 | 0.679630 |
| **SVM (RBF)** | 0.417890 | 0.628846 | 0.592593 |
| **Gaussian Process** | **0.866492** | 0.844231 | **0.914815** |
| **Random Forest** | 0.634347 | 0.650000 | 0.762963 |
| **Neural Network** | 0.841929 | **0.863386** | 0.883333 |
| **Naïve Bayes** | 0.525367 | 0.388499 | 0.579630 |
| **AdaBoost** | 0.504647 | 0.550528 | 0.648148 |

Table S3: Evaluation of different classifiers for standing exercises 1 and 2.

| **model** | **accuracy (macro-average)** | | |
| --- | --- | --- | --- |
|  | **Total Score** | **component 1** | **component 2** |
| **kNN** | 0.788225 | 0.763095 | 0.825978 |
| **SVM (linear)** | 0.618017 | 0.508333 | 0.561778 |
| **SVM (RBF)** | 0.804518 | 0.795238 | 0.806279 |
| **Gaussian Process** | **0.853061** | **0.826190** | **0.855458** |
| **Random Forest** | 0.550922 | 0.604762 | 0.585267 |
| **Neural Network** | 0.614555 | 0.614286 | 0.613215 |
| **Naïve Bayes** | 0.563365 | 0.579762 | 0.564815 |
| **AdaBoost** | 0.439628 | 0.464286 | 0.501902 |

Table S4: Evaluation of different classifiers for standing exercise 3 (progressions 0 and 1).

| **Model** | **accuracy (macro-average)** | | | | |
| --- | --- | --- | --- | --- | --- |
|  | **Total Score** | **component 1** | **component 2** | **component 3** | **component 4** |
| **kNN** | **0.911667** | 0.897667 | 0.903333 | **0.892333** | 0.832000 |
| **SVM (linear)** | 0.81245 | 0.76321 | 0.86254 | 0.77652 | 0.8965 |
| **SVM (RBF)** | 0.78656 | 0.56984 | 0.784112 | 0.69875 | 0.69771 |
| **Gaussian Process** | 0.897667 | **0.908500** | **0.916000** | 0.889000 | 0.828000 |
| **Random Forest** | 0.881667 | 0.894000 | 0.884000 | 0.917833 | **0.896000** |
| **Neural Network** | 0.850000 | 0.894333 | 0.896326 | 0.887167 | 0.919875 |
| **Naïve Bayes** | 0.851000 | 0.782833 | 0.806333 | 0.794833 | 0.905689 |
| **AdaBoost** | 0.528000 | 0.715167 | 0.444000 | 0.515667 | 0.556000 |

Table S5: Evaluation of different classifiers for standing exercise 3 (progression 2).

| **model** | **accuracy (macro-average)** | | | | |
| --- | --- | --- | --- | --- | --- |
|  | **Total Score** | **component 1** | **component 2** | **component 3** | **component 4** |
| **kNN** | 0.85698 | 0.873392 | 0.873684 | 0.873684 | **0.91215** |
| **SVM (linear)** | **0.873684** | 0.878655 | 0.872367 | 0.876272 | 0.892421 |
| **SVM (RBF)** | 0.442398 | 0.484503 | 0.442398 | 0.442398 | 0.305263 |
| **Gaussian Process** | 0.871947 | **0.889474** | 0.878947 | 0.878947 | 0.88241 |
| **Random Forest** | 0.844211 | 0.878947 | 0.884211 | **0.884912** | 0.884211 |
| **Neural Network** | 0.828947 | 0.884211 | 0.889474 | 0.821053 | 0.87241 |
| **Naïve Bayes** | 0.842343 | 0.878655 | **0.900246** | 0.85404 | 0.89321 |
| **AdaBoost** | 0.821053 | 0.863158 | 0.821053 | 0.821053 | 0.862154 |

Table S6: Evaluation of different classifiers for standing exercise 3 (progression 3).

| **model** | **accuracy (macro-average)** | | | | |
| --- | --- | --- | --- | --- | --- |
|  | **Total Score** | **component 1** | **component 2** | **component 3** | **component 4** |
| **kNN** | 0.861111 | 0.850585 | 0.829240 | **0.855556** | **0.888889** |
| **SVM (linear)** | 0.888889 | 0.872807 | 0.834503 | 0.794444 | 0.850000 |
| **SVM (RBF)** | 0.572222 | 0.587135 | 0.576316 | 0.561111 | 0.538889 |
| **Gaussian Process** | 0.488889 | 0.545614 | 0.552339 | 0.638889 | 0.672222 |
| **Random Forest** | **0.905556** | 0.845322 | 0.847076 | 0.888889 | 0.811111 |
| **Neural Network** | 0.866667 | 0.889474 | **0.878655** | 0.850568 | 0.872222 |
| **Naïve Bayes** | 0.883333 | 0.878655 | 0.812865 | 0.822222 | 0.711111 |
| **AdaBoost** | 0.883333 | **0.896491** | 0.715205 | 0.711111 | 0.755556 |

Table S7: Evaluation of different classifiers for standing exercise 4.

| **model** | **accuracy (macro-average)** | | | |
| --- | --- | --- | --- | --- |
|  | **Total Score** | **component 1** | **component 2** | **component 3** |
| **kNN** | 0.791323 | 0.784910 | 0.764640 | **0.799270** |
| **SVM (linear)** | 0.717710 | 0.589189 | 0.686261 | 0.691508 |
| **SVM (RBF)** | 0.576316 | 0.554054 | 0.583784 | 0.601905 |
| **Gaussian Process** | **0.918137** | **0.861111** | **0.883709** | 0.794048 |
| **Random Forest** | 0.762660 | 0.631156 | 0.723949 | 0.691508 |
| **Neural Network** | 0.849431 | 0.816967 | 0.856532 | 0.763175 |
| **Naïve Bayes** | 0.675818 | 0.621622 | 0.691066 | 0.649127 |
| **AdaBoost** | 0.639260 | 0.439715 | 0.605105 | 0.505635 |

Table S8: Evaluation of different classifiers for walking exercise 1.

| **model** | **accuracy (macro-average)** | | | |
| --- | --- | --- | --- | --- |
|  | **Total Score** | **component 1** | **component 2** | **component 3** |
| **kNN** | 0.854310 | 0.803704 | 0.785057 | 0.856207 |
| **SVM (linear)** | 0.69743 | 0.745345 | 0.632543 | 0.701537 |
| **SVM (RBF)** | 0.669874 | 0.77457 | 0.56977 | 0.569443 |
| **Gaussian Process** | 0.826724 | **0.814815** | 0.764023 | 0.664023 |
| **Random Forest** | **0.8996554** | 0.807407 | **0.845862** | **0.924713** |
| **Neural Network** | 0.446552 | 0.529630 | 0.502874 | 0.528966 |
| **Naïve Bayes** | 0.629064 | 0.551852 | 0.580690 | 0.665977 |
| **AdaBoost** | 0.865517 | 0.803704 | 0.685172 | 0.871264 |

Table S9: Evaluation of different classifiers for walking exercises 2 and 3.

| **model** | **accuracy (macro-average)** | | | | | | |
| --- | --- | --- | --- | --- | --- | --- | --- |
|  | **Total Score** | **component 1** | **component 2** | **component 3** | **component 4** | **component 5** | **component 6** |
| **kNN** | **0.813763** | **0.741592** | 0.700000 | 0.677778 | **0.712722** | 0.727451 | **0.745281** |
| **SVM (linear)** | 0.69854 | 0.569464 | **0.745125** | 0.698745 | 0.587452 | 0.658745 | 0.675412 |
| **SVM (RBF)** | 0.7755 | 0.658745 | 0.56547 | **0.782324** | 0.7214659 | **0.74698** | 0.710644 |
| **Gaussian Process** | 0.715385 | 0.659347 | 0.535417 | 0.592222 | 0.507137 | 0.694118 | 0.718027 |
| **Random Forest** | 0.703092 | 0.624449 | 0.616667 | 0.552899 | 0.650044 | 0.613725 | 0.695323 |
| **Neural Network** | 0.562707 | 0.579551 | 0.568750 | 0.398841 | 0.465736 | 0.615686 | 0.652423 |
| **Naïve Bayes** | 0.556750 | 0.500000 | 0.358333 | 0.387633 | 0.354122 | 0.425490 | 0.389158 |
| **AdaBoost** | 0.484125 | 0.426531 | 0.552083 | 0.477729 | 0.493927 | 0.227451 | 0.516709 |

| 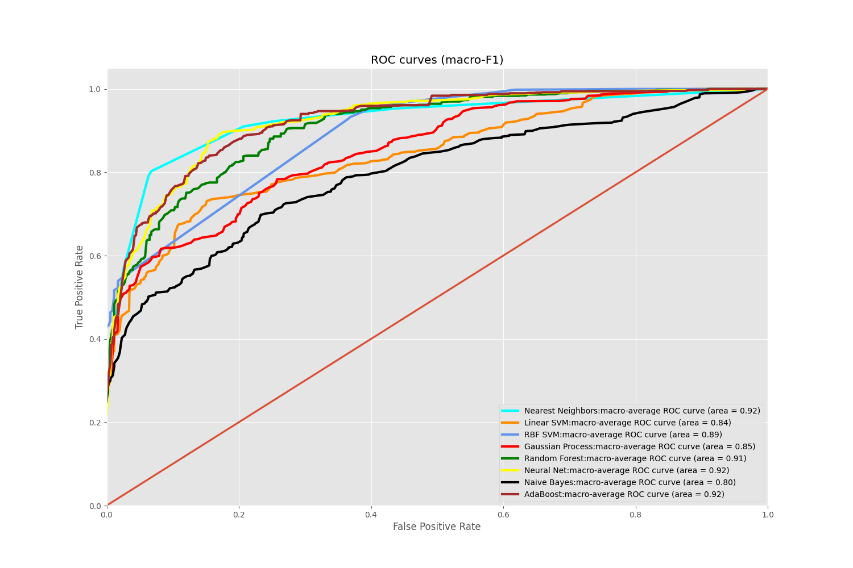  (a) | 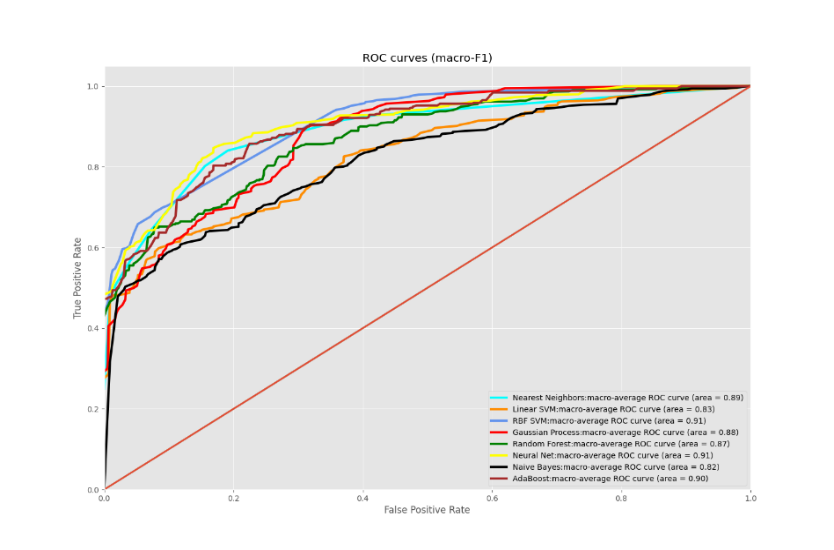  (b) |
| --- | --- |
| 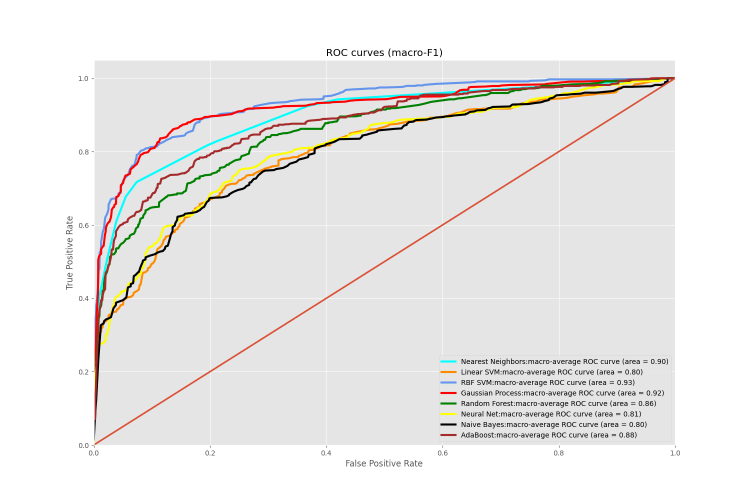  (c) | 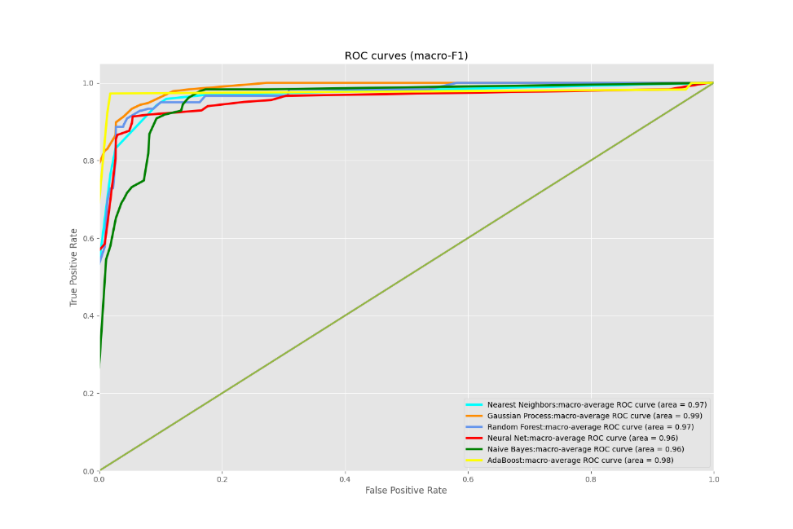  (d) |
| 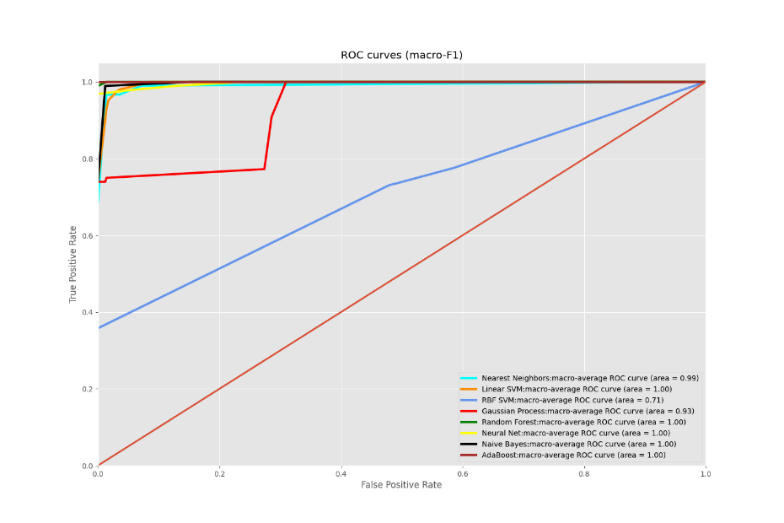  (e) | 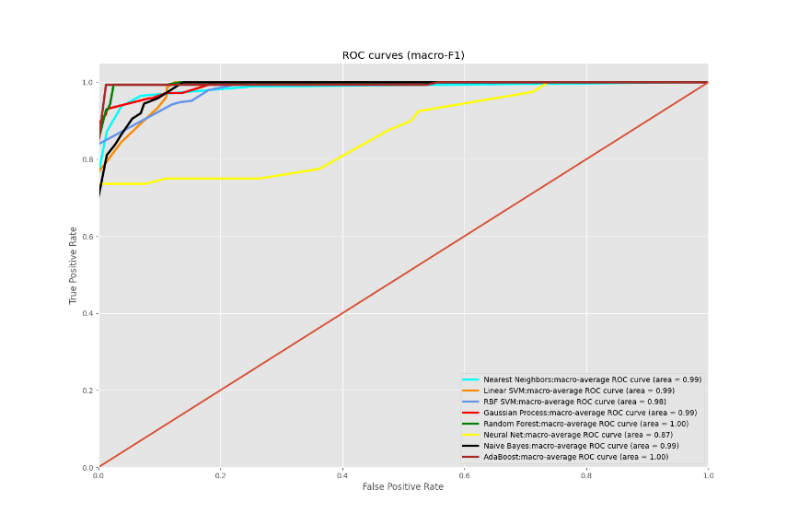  (f) |
| **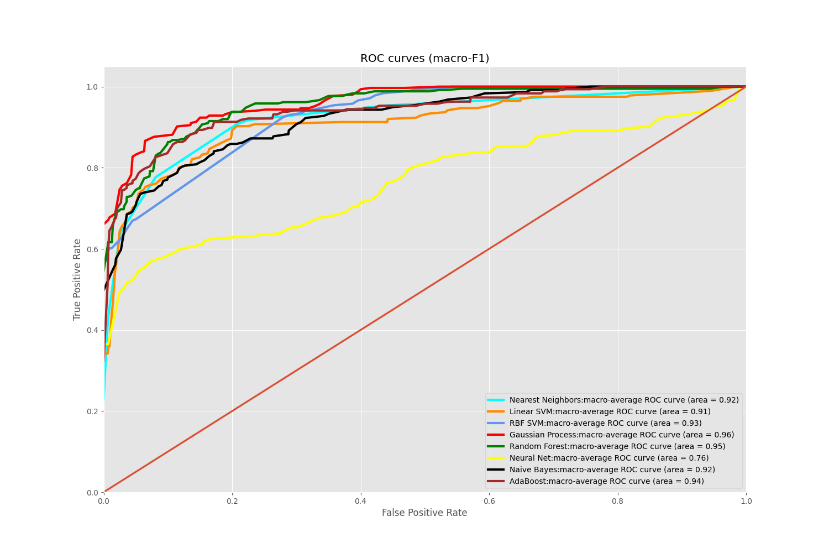**  (g) | **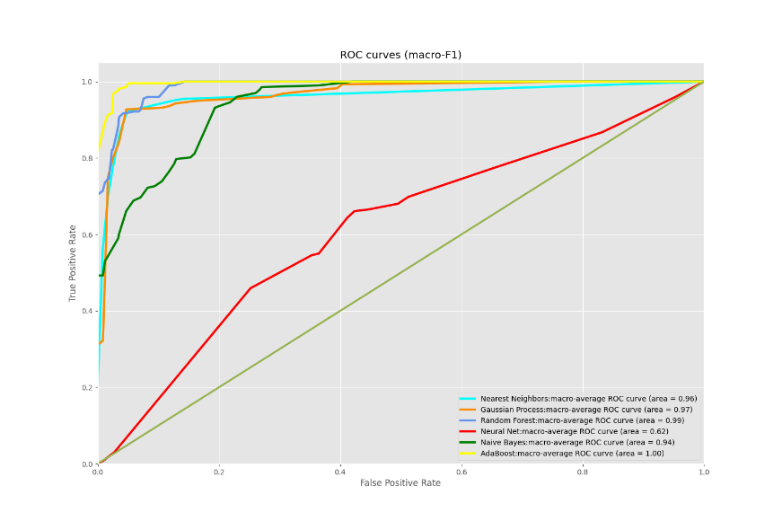**  (h) |
| **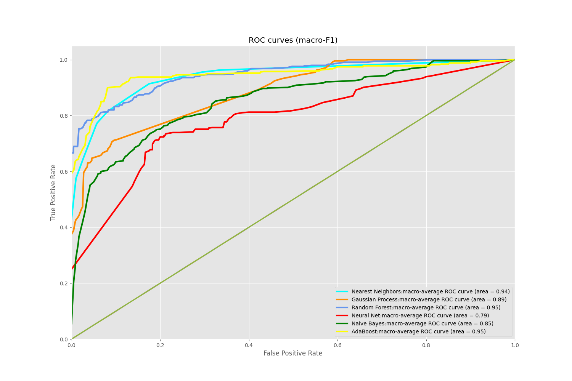**  (i) | |

Figure S2: ROC analysis on the Total score for (a) sitting exercises 1 and 2, (b) sitting exercise 3, (c) standing exercises 1 and 2, (d) standing exercise 3 (progressions 0 and 1), (e) standing exercise 3 (progression 2), (f) standing exercise 3 (progression 3), (g) standing exercise 4, (h) walking exercise 1 and (i) walking exercises 2 and 3.
